# Measles outbreaks in displaced populations: a review of transmission, morbidity and mortality associated factors

**DOI:** 10.1186/1472-698X-10-5

**Published:** 2010-03-19

**Authors:** Isidore K Kouadio, Taro Kamigaki, Hitoshi Oshitani

**Affiliations:** 1 Department of Virology, Tohoku University Graduate School of Medicine, Sendai, Japan

## Abstract

**Background:**

Measles is a highly contagious infectious disease with a significant public health impact especially among displaced populations due to their characteristic mass population displacement, high population density in camps and low measles vaccination coverage among children. While the fatality rate in stable populations is generally around 2%, evidence shows that it is usually high among populations displaced by disasters. In recent years, refugees and internally displaced persons have been increasing. Our study aims to define the epidemiological characteristics and risk factors associated with measles outbreaks in displaced populations.

**Methods:**

We reviewed literature in the PubMed database, and selected articles for our analysis that quantitatively described measles outbreaks.

**Results:**

A total of nine articles describing 11 measles outbreak studies were selected. The outbreaks occurred between 1979 and 2005 in Asia and Africa, mostly during post-conflict situations. Seven of eight outbreaks were associated with poor vaccination status (vaccination coverage; 17-57%), while one was predominantly due to one-dose vaccine coverage. The age of cases ranged from 1 month to 39 years. Children aged 6 months to 5 years were the most common target group for vaccination; however, 1622 cases (51.0% of the total cases) were older than 5 years of age. Higher case-fatality rates (>5%) were reported for five outbreaks. Consistent factors associated with measles transmission, morbidity and mortality were vaccination status, living conditions, movements of refugees, nutritional status and effectiveness of control measures including vaccination campaigns, surveillance and security situations in affected zones. No fatalities were reported in two outbreaks during which a combination of active and passive surveillance was employed.

**Conclusion:**

Measles patterns have varied over time among populations displaced by natural and man-made disasters. Appropriate risk assessment and surveillance strategies are essential approaches for reducing morbidity and mortality due to measles. Learning from past experiences of measles outbreaks in displaced populations is important for designing future strategies for measles control in such situations.

## Background

Measles is an acute viral disease caused by a paramyxovirus of the genus *Morbillivirus*. Symptoms include fever, cough, runny nose, red eyes and a generalized maculopapular erythematous rash. It is spread by respiratory system contact with fluids from an infected person's nose and mouth by either droplet (coughing or sneezing) or aerosol transmission. Although a vaccine has been available since 1959 [1], measles remains an important cause of morbidity and mortality in children, particularly in developing countries where more than 95% of measles-associated deaths occur [2-4]. Measles vaccination efforts have achieved major public health gains, resulting in a 74% decline in measles deaths worldwide between 2000 and 2007 from an estimated 750, 000 to 197, 000, with a decline of about 90% in the eastern Mediterranean and sub-Saharan African regions [5].

Measles is an important public health concern during disasters involving massive population displacements who end up living in camps [6]. The World Health Organization (WHO) recognizes refugees as one of the high-risk groups for measles outbreaks. Several outbreaks have been reported among refugees and other emergency settings [7-9] due to their characteristic massive population displacements, overcrowding, high population densities and low vaccination coverage [10,11]. Overcrowding is associated with the transmission of higher infectious doses of measles virus, resulting in more severe cases of clinical disease [12], which makes measles more often the leading cause of mortality among children in refugee populations.

Emergency relief operations are often conducted during situations of widespread famine and massive population displacements in overcrowded settings, where serious problems of acute and chronic undernutrition prevail, particularly among children younger than 5 years of age [13]. Under such circumstances, immunization programmes have sometimes been implemented late, slowly or not at all. Thus, preventable outbreaks of measles have commonly occurred in refugee camps.

Health or nutritional status is usually assessed in three ways: by measuring growth and body composition (anthropometric indicators); by analyses of biochemical contents of blood and urine (biochemical indicators) and by clinical examinations for external physical signs of nutrient deficiencies (clinical indicators). The anthropometric measurements are common easy ways to assess health and nutrition status including variables such as age, weight, height and gender to calculate three indices, namely, weight for age, height for age and weight for height. For decision making in refugee settings, weight for height surveys or screenings are the most efficient strategies for nutritional data collection [14]. The National Center for Health Statistic (NCHS) population has been the reference most commonly used in national programs for individual growth monitoring and generating population-based estimates of child malnutrition [15] and in emergency settings to determine admission to and discharge from feeding programs [16]. This standard reference malnutrition status for cases of less than 80% weight for height.

Despite measles control strategies in refugee settings [17,18], case-fatality rates (CFR) as high as 34% have been reported. In contrast, measles fatality rates in stable populations are around 2% [19,20]. Among refugees and internally displaced populations, WHO and United Nations Children's Fund (UNICEF) recommend vaccinating children aged 6 months to 14 years, with coverage exceeding 95%. However, specific target age groups during such campaigns should be determined based on the local epidemiology of the disease [17]. They also recommend that improvements in case management and surveillance should be made to reduce morbidity and mortality associated with this disease. Vaccinating children to prevent measles is considered a cost effective priority in displaced populations living in camps [11,21].

Natural disasters as well as complex emergencies can lead to the displacement of a large number of people either within the same country (internally displaced persons; IDP's) or into another country (refugees). Complex emergencies are situations in which mortality among the civilian population substantially increases above the population baseline, either as a direct result of war or indirectly through increased prevalence of malnutrition and/or transmission of communicable diseases, particularly if the latter results from deliberate political and military policies and strategies. This definition does not include natural disasters that are usually short term catastrophic events of atmospheric, geologic and hydrologic origins, which necessitate a qualitatively different response.

According to the United Nations High Commissioner for Refugees (UNHCR), the number of refugees and IDP's has increased to 15.2 million and 26 million, respectively, at the end of 2008 due to various reasons such as natural disasters and internal conflicts [22]. In such settings, health systems often collapse or become non-functional. In addition, overcrowding, high population density in rudimentary shelters or camps, inadequate safe water and sanitation and poor vaccination status among refugees increase the risk of infectious disease spread [23] and result in higher morbidity and mortality, especially among children.

Surveillance is the ongoing systematic collection, analysis and interpretation of health data essential for planning, implementation and evaluation of health practices. It is closely integrated with the timely dissemination of these data to those who need them. Surveillance can be passive or active.

Passive surveillance is health facility-based surveillance that is normally established in each camp. Active surveillance is house-to-house case findings that can be conducted among refugee families, by involving community leaders and health workers. It increases the potential to detect cases during an early phase for timely intervention. Both surveillance types can be combined, but this increases the demand for additional human resources and equipment. Applying surveillance data to disease prevention and control requires a functional capacity for data collection, analysis and dissemination with a link to the public health program [24]. The objective of surveillance is to provide information on a regular basis for use in decision making.

In the context of displaced populations, three main reasons highlight the need for implementing an appropriate surveillance system as an extension of the initial assessment just before any intervention [21]. These include the extreme vulnerability to epidemics, malnutrition or other acute health problems; the sudden changes that can occur during an emergency phase within the population itself (size and composition) as well as the health conditions (health background) and the need to have quantitative data as base information on refugee situations for communicating to other partners, and possibly, to the media and/or donors.

The high morbidity and mortality due to measles in displaced populations will be significantly reduced if an appropriate surveillance system and control measures are implemented in a timely and coordinated manner. Based on epidemiological analysis, appropriate public health measures were only introduced to disaster and complex emergencies in the late 1970s [25,26]. Since then, studies and guidelines on measles control in such settings have been well documented [6,26-29]. However, there are only a few published studies reporting complete epidemiological investigations on outbreaks associated with the disease during disasters and among displaced populations. In this study, we review the epidemiology of measles in disaster and refugee situations over 26 years. In addition, we describe some important aspects of measles control to be considered by public health and humanitarian workers.

## Methods

**Figure 1:**
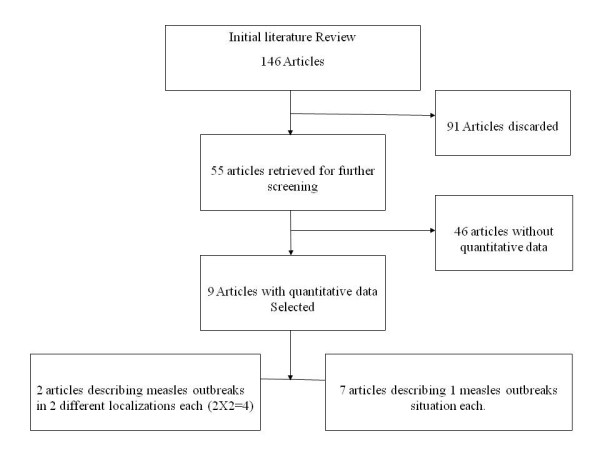
**Process flow for the review of the literature**.

We conducted a review of published articles on measles outbreaks in population displaced by disasters and summarized the potential risk factors involved in the transmission, morbidity and mortality of measles in these settings. Literature searches were conducted using the PubMed database, and were limited to articles written in English. Searches were conducted from October 2007 to March 2008 using various combinations of the following search terms: (Natural disaster OR armed conflict OR complex emergencies OR refugees OR IDP's) AND (measles OR epidemiology OR outbreak OR surveillance). We used all the 146 articles that we were initially aware of to describe measles outbreaks in population displaced by disasters. A team of two individuals (IKK and TK) independently screened the titles and abstracts of each citation and identified all citations for full review when there was any possibility that the study contained any description of measles outbreak in population displaced by disasters we were interested in. This screening process yielded 55 full text original publications identified by one or both (IKK and TK) (Figure 1). We masked the results of all publication selected for full review by obscuring them with a black marker from the tables and text. The third author (OH), an expert in the field, evaluated independently each masked article to determine eligibility. Our agreement on studies evaluated within the team was good and all disagreements were solved by consensus which required individuals to discuss the reasoning for their decisions. We included original articles that described measles outbreaks in population displaced by disasters, with quantitative data. We excluded measles outbreaks studies that were not associated with disasters situations and those without quantitative data. Finally, nine articles matching these requirements were used for analysis (Table 1). If an article reported two separate situations analysis matching our criteria, we included their results from the analysis with the most appropriate adjustment. If an article reported two measles situation analysis in disaster and non disaster settings, the one that is described in disaster settings was included in our study. The measles outbreaks not following disaster situation was excluded as a potential confounder. Therefore we agreed to investigate the measles outbreaks situation analysis described in post-disaster situations. We assessed the following characteristics generated in an established questionnaire with a personal coding system. The following informations were collected: (1) nature and impact of the disaster on the displaced populations, including the type and year of the disaster, demographic characteristics of the displaced populations, their country of origin and asylum; (2) surveillance for measles, including the presence and type of surveillance, and documentation on laboratory investigation; (3) epidemiological characteristics of measles outbreak, including the number, gender and age range of cases, the attack rate (AR), the number of deaths with case-fatality rate and (4) characteristics of measles vaccination, including the vaccination status before entering camps, the target population for vaccination campaigns and the vaccination coverage for the campaigns. Before carrying out the analysis we, we specified several hypotheses to test potential explanations for variability (I.e heterogeneity) among the studies. We listed-up all the potential risk factor for measles morbidity and mortality from each selected articles and numbered them. We pooled them randomly and categorized them with their reference source. The data generated from the questionnaire were recorded using Microsoft Excel 2000 (Microsoft, USA) and analyzed with Epi-Info version 6 (Centers for Disease Control and Prevention, USA) software.

**Table 1:** List of the 9 selected articles and dates of publication

Nb	Titles of selected articles	Year of Publication
1	Cambodian disaster relief: refugee camp medical care[37]	1982

2	Epidemiological assessment of health and nutrition of Ethiopian refugees in emergency camps in Sudan 1985[20]	1987

3	Measles outbreaks in the Mozambican refugee camps in Malawi: the continued need for an effective vaccine[7]	1990

4	Measles in Vietnamese refugee children in Hong Kong [8]	1999

5	An outbreak of measles in Tanzanian refugee camps [11]	2003

6	Measles mortality among Afghan refugees' children [4]	2002

7	Retrospective measles outbreak investigation: Sudan, 2004[56]	2006

8	Emergency measles control activities-Darfur, Sudan, 2004[47]	2004

9	Measles transmission following the tsunami in a population with high one-dose vaccination coverage, Tamil Nadu, India[3]	2006

## Results

### Literature Review and Data Screening

Although 146 articles were retrieved using the selected keywords, only 55 (37.7%) actually reported on measles outbreaks in displaced populations (Figure 1). After further review, only nine articles containing consistent quantitative data were selected. Among these nine articles, two described measles outbreaks following disasters in two different situation analyses respectively. Finally, we focused our analysis on 11 measles outbreaks that occurred from 1979 to 2005 (Table 2).

**Table 2:** Documented 11 studies reviews (from the 9 selected articles) on disasters and measles outbreaks in displaced populations, 1979--2005

Countries, type of displaced population/Reference	Type of disaster/Year	Total displaced populations	Documentation surveillance approach	AR (Case/Pop. at Risk)	CFR (Death/Cases)	Documentation on laboratory confirmation	Documentation on malnutrition
Cambodian refugees in Thailand[37]	Conflict 1979--1980	Average: 53666 (Pop. in camp)	Not described	(161/924) 17.4%	(10/161) 6.2%	No	Yes

Ethiopian refugees in Wad Kowli (Sudan) [20]	Conflict + Famine 1985	85000 (Pop. in camp)	Not described	(2639/85000) 3.1%	(855/2639) 32.4%	Not described	Yes

Mozambican refugees in Malawi [7]	Conflict 1988--1989	78568 (Pop. in camp)	Passive surveillance	(744/78568) 0.95%	(103/744) 13.8%	Not described	No

Vietnamese refugees in Hong Kong [8]	Conflict 1991--1992	7017 (Pop. in camp)	Passive surveillance	(262/1026) 25.5%	(2/262) 0.76%	Not described	Yes

Burundian refugees in Tanzania [11]	Conflict 2000--2001	170500 (pop. in camp)	Passive surveillance + Active case findings	(1062/170500) 0.62%	(0/1062) 0.0%	Yes	No

Turkmen + Uzbek refugees in Afghanistan [4]	Conflict 2000--2001	115000 (Pop. in camp)	Not described	(80/22000) 0.36%	(12/80) 15.0%	Yes	Yes

IDP's in White Nile state (Sudan) [56]	Conflict 2003--2004	91000 (Total Pop. of administrative unit)	Active case findings	(621/91000) 0.68%	(8/621) 1.3%	Yes	Yes

IDP's in Khartoum (Sudan)[56]	Conflict 2003--2004	75000 (Pop. in administrative unit)	Active case findings	(523/75000) 0.69%	(2/523) 0.4%	Yes	Yes

IPD's in North Darfur (Sudan) [47]	Conflict 2004	657774 (Pop. in targeted zone)	Passive surveillance	(521/512058) 0.10%	(88/521) 16.9%	Yes	Yes

IDP's in West Darfur (Sudan) [47]	Conflict 2004	688984 (Pop. in targeted zone)	Passive surveillance	(204/461619) 0.04%	(20/204) 9.8%	Yes	Yes

IDP's in Tamil Nadu (India) [3]	Tsunami 2004--2005	53104 (Total Pop. in the affected villages)	Passive surveillance + Active case findings	(71/53104) 0.13%	(0/71) 0.0%	Yes	No

IDP's = internally displaced persons AR = attack rate CFR = case-fatality rate

### Demographic Characteristics of the Populations

The outbreaks reviewed were reported from Africa and Asia, mostly in post-conflict settings. In the overall displaced populations, the number of IDP's (1,565,862) was more than twice the number of refugees (509,751). The characteristics of the populations at risk (denominators) to measles among investigations were variable. The smallest populations were Cambodian refugees at a camp hospital in Thailand (924) and Vietnamese refugee children at a camp hospital in Hong Kong (1026). The largest populations comprised Mozambican refugees in Tanzanian camps (170,500) followed by Ethiopian refugees in Wad Kowli camps (85,000). The observation period was also variable among the studies, with the shortest period of 10 days (Ethiopian refugees in Wad Kowli camps in Sudan) and the longest observation period of 14 months (Burundian refugees in Tanzanian camps). Malnutrition, (weight for height < 80% of the NCHS reference median) was described in eight of the 11 outbreaks reviewed. The malnutrition rate reviewed was important among Ethiopian refugees in Sudan, with the toll reaching 52% of which 20% were severe.

### Surveillance

A surveillance approach to detect and report measles cases was described in eight of the 11 outbreaks (Table 2). Passive surveillance alone was most frequently described (four outbreaks), while active case findings alone were implemented during two outbreaks. A combined approach of passive and active surveillance was described during two outbreaks (tsunami victims in India and Burundian refugees in Tanzanian camps). The case definition of measles was mentioned in six of the 11 outbreaks. All of these followed the WHO case definition, which is the occurrence of generalized rash and high-grade fever (38°C or higher) and any of the following: cough, coryza (inflammation of mucous membranes lining the nasal cavity) or conjunctivitis. Laboratory tests to confirm measles were performed during seven outbreaks, but none mentioned the quality of laboratory surveillance.

### Measles Morbidity

The total number of cases varied from 71 (tsunami victims in India) to 2639 (Ethiopian refugees in Sudan) per outbreak (mean = 625; median = 521). The attack rate (AR) was defined as the number of measles cases per population at risk in each setting. The population at risk included different subsets of the total population. Therefore, the AR could not be compared between outbreaks due to differences in population subsets.

Vietnamese refugees admitted to a camp hospital in Hong Kong had the highest AR of 25.5%, followed by Cambodian children (AR = 17.4%) admitted to hospital Ward 9 in the Thailand camp (Table 2). However, in these studies, only a small subset of the population (i.e. children in camp hospitals) was used as the population at risk. Among the studies that included the total camp population as the population at risk, the highest AR was described for Ethiopian refugees in Sudanese camps (AR = 3.10%). The average male to female ratio was 1.08. Specifically, higher male to female ratios were noted among Cambodian refugees in camp hospitals (ratio = 1.30) and Vietnamese refugee children (ratio = 1.45). The overall age range of the cases was 1 month to 39 years. The most affected age group was children aged 5-15 years in five of the eight outbreaks investigated (Table 3).

**Table 3:** Measles cases not belonging to the target populations during respective vaccination campaigns from available data

		Most affected age groups					
					
Countries, type of displaced population/Reference	Vaccination status	<5 years old	5--15 years old	15--39 years old	Population target age for vaccination	Younger than population target age	Older than population target age
Cambodian refugees in Thailand[37]	-	130/161(81%)	31/161 (19%)	0	<5 years old children	-	31/161 (19%)

Ethiopian refugees in Wad Kowli (Sudan) [20]	-	1232/2759 (46.7%)	1407/2639 (53.2%)	120/2759 (4.45%)	6 months--5 years old	-	-

Mozambican refugees in Malawi [7]	17%	426/744 (57.3%)	318/744 (42.7%)	0	6 months--5 years old	85/744 (11.4%)	318/744 (42.7%)

Vietnamese refugees in Hong Kong [8]	Described to be low	262/262 (100%).	0%	0%	6 months--12 years old	10/262 (3.8%)	-

Burundian refugees in Tanzania [11]	Described to be low	511/1062 (48%)	551/1062 (52%)	0	6 months--5 years old	-	551/1062 (52%)

Turkmen + Uzbek refugees in Afghanistan [4]	-	-	-	-	-	-	-

IDP's in White Nile state (Sudan) [56]	46%	285/621 (45.9%)	307/621 (49.4%)	29/621 (4.67%)	<5 years old	-	336/621 (54%)

IDP's in Khartoum (Sudan) [56]	52%	163/523 (31.2%)	298/523 (57%)	62/523 (18.9%)	<5 years old	-	360/523 (68.8%)

IPD's in North Darfur (Sudan) [47]	46%	-	-	-	9 months--15 years old	-	-

IDP's in West Darfur (Sudan) [47]	57%	-	-	-	9 months--15 years old	-	-

IDP's in Tamil Nadu (India) [3]	95%	31/71 (43.7%)	40/71 (56%)	0%	6 months--5 years old	-	26/71 (36.2%)

Total		3040/6203 (49.0%)	2952/6203 (47.6%)	211/6302 (3.4%)		95/1006 (9.4%)	1622/3182 (51.0%)

IDP's = internally displaced persons

### Measles Mortality

The number of deaths also varied from 0 to 855 (mean = 100; median = 10). The highest CFR were reported among Ethiopian refugees in Wad Kowli camps in 1985 (32.4%), followed by IDP's in inaccessible areas in North Darfur in 2004 (16.9%; Table 2). As a general observation, passive surveillance alone was associated with higher case fatality, active surveillance alone with low fatality and no fatalities were reported for the tsunami victims in India and the Burundian refugees in Tanzanian camps, where combined passive and active surveillance were implemented. The CFR prior to 2000 was higher (mean = 13.1; median = 10) compared to that after 2000 (mean = 6.2; median = 1.3). Information regarding vaccination status was reported for eight outbreaks and most of them were in context where a one-dose strategy for measles was implemented. Out of eight outbreaks seven occurred in poorly vaccinated populations prior to arrival in camps (vaccination coverage: 17-57%). Only the measles outbreak among the tsunami victims occurred despite a prior coverage of 95% (Table 3). The target age group for vaccination campaigns was described in 10 of the 11 outbreaks. Children aged 6 months to 5 years were the most common target group for vaccination, but a total of 1622/3182 (51%) measles cases were older (>5 years old). Only 9.44% (95/1006) were younger than the target age for vaccination (Table 3).

### Summary of Contributing Factors for Measles Morbidity and Mortality

Table 4 summarizes the potential risk factors for measles transmission, mortality and morbidity that we identified from the reviewed articles. In general, the described risk factors can be categorized into risk factors related to vaccination status, living conditions, movements of refugees, nutritional status and effectiveness of control measures including vaccination campaigns, surveillance and security situations in the affected zones.

**Table 4:** Review of contributing risk factors for measles Morbidity and Mortality in displaced populations, 1979--2005

	Potential risk factors involved	Possible solutions
1	Overcrowded and high density camps [4,8,20,37]	Improve camp planning and shelter

2	Poor vaccination status due to the continuous arrival of new refugees in camps [4,7,8,11,47,56]	Vaccination of refugees on arrival in camps

3	Poor vaccination status in the surrounding community [11]	Coordination of vaccination campaign in camps and in the surrounding community in collaboration with local public health authorities.

4	Narrow target age group for vaccination campaign [7,8,11,20,37,47]	Extension of population target to >15 years old

5	Primary vaccination failure due to vaccination in lower age group [7,8]	Revaccinate at 9 months

6	Lower vaccination coverage in high risk group [11]	Increase coverage in high risk age group guided by good quality of surveillance data

7	One time measles vaccination strategy [3]	Routine vaccination plus supplemental vaccination

8	Frequent visit of refugees between camps [3,8]	Restriction of refugees movement during outbreak period

9	Frequent movement of refugees in the neighbouring community and in other camps [3,11]	Restriction measures for visit from surrounding communities during outbreak period

10	Malnutrition and famine [4,8,20,37,47,56]	Consideration of vitamin A provision

11	Insecurity and inaccessibility to target zones [56]	Re-establish the routine vaccination service after the security situation is normalized.

12	No or limited surveillance system [4,37,47]	Improving surveillance approach for early case detection

13	Lack of laboratory confirmation of suspect cases[35]	Establishing appropriate laboratory testing

14	Association with other diseases [20,37]	Implementation of integrated management of childhood illness (IMCI)

IMCI = integrated management of childhood illness

## Discussion

### Geographical Characteristics of Disasters and Measles

Measles outbreaks had occurred among populations displaced by disasters in Africa and Asia where low routine vaccination coverage, even for the general population, had been reported [30]. Almost all measles outbreaks (10/11; 91.0%) analyzed in the present study occurred in post-conflict situations with a collapse of the health system and disruption of the immunization program. Therefore, no prior measles vaccination campaign was documented for any of the outbreaks following conflict situations in our study. According to the WHO and UNICEF, during the 1990s, at least seven of the 15 countries that reported low vaccination coverage of <50% were affected by internal conflicts. They also described large outbreaks of measles, primarily in countries affected by internal conflicts [6]. Importation of the measles virus from refugee camps in Africa (Kenya) into developed countries due to the movement of refugees was also described [31]. Previous publications indicated that 2/3 of the total deaths in post-conflict situations were attributable to communicable diseases [26,32].

### Risk Factors and Characteristics of Patients

Disasters contribute to increased population displacements and densities in camps [33], creating new conditions for the spread of infectious diseases [34-36]. The highest incidence rate of 25.5% was described for Vietnamese children in Hong Kong camps where housing was cramped, consisting of large huts that housed approximately 250 refugees on three tier bunk beds [37]. The affected age group from 1 month to 39 years was described in our study and those aged from 5 to 15 years were the most affected in five of the eight investigations. Previous studies have also reported similar age patterns for measles in older age groups [38,39]. Infection in an older age group may have been due to the absence of exposure and/or the failure of previous measles vaccination campaigns. Measles infection in a younger group (<6 months) in our study was probably due to low maternal antibody levels, as described in the published literature [40-43].

High CFR of >5% were described in six of the nine outbreaks reviewed. These were significantly higher than that of 2% in stable populations [44]. The highest fatality rate of 32.4% was described among Ethiopian refugees in Sudanese camps where a war conflict occurred in context of widespread famine with malnutrition rates as high as 32% (with 9-20% severe malnutrition) [20]. Previous publications have documented an association between severe malnutrition and high measles case-fatality rates [19,29,45]. Vitamin A supplements together with measles vaccination have been recommended in order to minimize mortality [33,46].

In other outbreaks, lack of basic surveillance or an inappropriate surveillance system compromised early case detection, and therefore, early response leading to an increase in mortality due to measles with the obvious limited access to health care. In northern Sudan, the inaccessibility of targeted areas for public health intervention, surveillance and vaccination campaigns due to the ongoing conflict may have indirectly contributed to the increase of risk factors for morbidity and mortality [47]. All of the outbreaks referred to in this study were in context where there were no two-dose vaccination strategies for measles, normally recommended by the WHO.

All post-conflict measles outbreaks occurred in conditions characterized by poor or no vaccination of the displaced populations prior to their arrival in the refugee camps. The only outbreak that occurred among those with a high coverage of 95% was the outbreak among the tsunami victims. It was suggested that the single-dose vaccination strategy may have been responsible for this outbreak [3,48]. Furthermore, other studies in Sri Lanka [49], Latin America [50] and Romania [51] reported similar failures of the single-dose vaccination strategy.

Several guidelines for measles vaccination in emergencies recommend that the primary targets for measles vaccination should be children aged 6 months to 5 years [6,52]. However, in total, 51.0% of the overall cases were older than the target age group for vaccination (>5 years) and may have remained unvaccinated with the possibility to sustain transmissibility in camp residents as well as in the continuous new arrivals of displaced populations in camps. This highlights the need to extend the population target according to local measles epidemiology.

Clinical diagnosis of measles from a number of conditions that present with fever and rash symptoms [7] is difficult without laboratory confirmation, mostly for dark-skinned people. Therefore, laboratory testing should be an essential component of surveillance to confirm measles infection in order to establish the appropriate intervention, including vaccination.

### Impact of the Surveillance Approach and Early Warning Response

It is noteworthy that there were no fatalities among the Burundian refugees in Tanzanian camps and among the tsunami victims in India. The absence of deaths in these outbreaks may have been due to the implementation of active case findings that supplemented the existing passive surveillance, which may have led to early case detection and implementation of rapid responses (case management or vaccination).

Regarding treatment, all children identified with clinical measles in refugee camps should be enrolled in a feeding program, and their nutritional status should be carefully monitored. Children with complications should be given a standard treatment (e.g. oral rehydration therapy for diarrhoea) and an antibacterial (penicillin or co-trimoxazol) against possible super-infection manifestations (pneumonia and middle ear infection) [53]. Increased intake of oral fluids and continued feeding should be routine in all cases of measles [46]. The ideal situation would be to vaccinate all of the displaced populations against measles at the time of their arrival in camps, preferably using the combined Measles-Mumps-Rubella (MMR) vaccines [54]. Specifically, this should be implemented in the absence of laboratory surveillance.

This implies making vaccine equipment, supply and trained personnel available as soon as the persons at risk gather in the camps. Furthermore, this should supplement existing passive surveillance with active case findings in outbreak investigations in order to increase early case detection and rapid response. The reality in the field is that these interventions pose logistical and economical challenges. Therefore, public health officers in the field have to prioritize interventions due to limited budgets.

Recently, measles vaccination efforts through the measles initiative program have reaped major public health gains, resulting in a 74% decline in measles deaths between 2000 and 2007 worldwide [55], which is in line with the fatality rate decline described after the year 2000 in our study. However, measles remains endemic in certain areas of the world. The responses to these measles outbreaks have remained generally constant over the time period in our reviewed articles. For the control of measles in displaced population settings, our study suggests:

- Vaccination campaign based on the local epidemiological factors

- The initial priority age group of 6 months to 5 years to be extended to 15 years and older according to budget allocations

- To increase coverage (>95%) in the target age group with a second opportunity to be given to children already vaccinated prior to 9 months.

- To prioritize measles vaccine together with vitamin A supplements for infected, malnourished children (100000 UI on day 1 and day 2 for children aged 6 months-12 years and 200000 UI on day 1 and day 2 for those aged >12 years).

- To vaccinate refugees upon arrival in camps

- To promote simultaneous vaccination campaigns within the refugee population in camps and the surrounding host community population in a common effort with the local health authorities.

- To improve surveillance by implementing community active case detection to supplement passive surveillance for early case detection for rapid response

- To improve case management by providing vitamin A and appropriate treatments for infectious complications associated with measles (pneumonia, severe diarrhoea, encephalitis and severe malnutrition).

- To monitor measles patients associated with malnutrition, and if necessary, to enrol them in a supplementary feeding program.

### Study Limitations

First, there were large variations in the settings with effects on the denominator populations as well as the periods of studies, which made it difficult to make comparisons between outbreaks. Second, because we restricted our search only to the PubMed database for English publications and published articles with quantitative data, there was a possibility of publication bias and lack of a broad information base given the absence of articles from the grey literature as well as other databases. With these limitations, our investigation may not provide a true picture of measles outbreaks in all displaced populations. However, given what we know from this study and for the purpose of improving measles control in displaced populations, we call for more research among displaced populations with subsequent publication of results.

## Conclusion

Measles is a significant public health concern in displaced populations. A number of risk factors may increase measles transmission, morbidity and mortality in populations displaced by disasters, including low vaccination status and coverage, lack of proper surveillance and response mechanisms and inadequate vaccination campaigns. Consideration of local measles epidemiology is important to establish vaccination priorities, as is the vaccination of age groups most at risk to confer adequate herd immunity. It is necessary to learn lessons from past experiences of measles outbreaks in displaced populations to develop future strategies and budget-based priority interventions towards measles control in post-disaster settings.

## Abbreviations

AR: Attack Rate; CFR: Case Fatality Rate; HO: Hitoshi Oshitani; IDP's: Internally Displaced Persons; IKK: Isidore Koffi Kouadio; IMCI: Integrated Management of Childhood Illness; NCHS: National Center for Health Statistics; MMR: Measles-Mumps-Rubella; TK: Taro Kamigaki; UNHCR: United Nations High Commissioner for refugees; UNICEF: United Nations Children's Fund; WHO: World Health Organization.

## Competing interests

The authors declare that they have no competing interests.

## Authors' contributions

IKK participated in the conceptual framing of the findings, analysis and writing subsequent drafts of the manuscript. TK assisted in the findings, analysis and revision of the manuscript. HO participated in the design, supervision of the study and contributed to the revision of the paper. All authors have read and approved the final manuscript

## Pre-publication history

The pre-publication history for this paper can be accessed here:

http://www.biomedcentral.com/1472-698X/10/5/prepub
